# Gibberellic Acid Initiates ER Stress and Activation of Differentiation in Cultured Human Immortalized Keratinocytes HaCaT and Epidermoid Carcinoma Cells A431

**DOI:** 10.3390/pharmaceutics13111813

**Published:** 2021-10-30

**Authors:** Mariya Vildanova, Polina Vishnyakova, Aleena Saidova, Victoria Konduktorova, Galina Onishchenko, Elena Smirnova

**Affiliations:** 1 Faculty of Biology, Lomonosov Moscow State University, Leninskie Gory, 1, Bld. 12, 119234 Moscow, Russia; aleena_gladkikh@mail.ru (A.S.); virgo584@yandex.ru (V.K.); galina22@mail.ru (G.O.); kinggobi@yandex.ru (E.S.); 2 National Medical Research Center for Obstetrics, Laboratory of Regenerative Medicine, Gynecology and Perinatology Named after Academician V.I. Kulakov of Ministry of Healthcare of Russian Federation, 4 Oparina Street, 117997 Moscow, Russia; p_vishnyakova@oparina4.ru; 3 Histology Department, Peoples’ Friendship University of Russia (RUDN University), 6 Miklukho-Maklaya Street, 117198 Moscow, Russia

**Keywords:** autophagy, differentiation, epidermoid carcinoma, ER stress, gibberellic acid, human keratinocytes, plant hormones

## Abstract

Diterpenoid plant hormone gibberellic acid (GA) plays an important role in regulation of plant growth and development and is commonly used in agriculture for activation of plant growth and food production. It is known that many plant-derived compounds have miscellaneous biological effects on animals and humans, influencing specific cellular functions and metabolic pathways. However, the effect of GA on animal and human cells remains controversial. We investigated the effect of GA on cultured human cell lines of epidermoid origin—immortalized non-tumorigenic keratinocytes HaCaT and carcinoma A431 cells. We found that at a non-toxic dose, GA upregulated the expression of genes associated with the ER stress response—*CHOP*, *sXBP1*, *GRP87* in both cell lines, and *ATF4* predominantly in A431 cells. We also showed that GA was more effective in upregulating the production of ER stress marker GRP78, autophagy marker LC3B-II, and differentiation markers involucrin and filaggrin in A431 cells than in HaCaT. We conclude that GA induces mild ER stress in both cell lines, followed by the activation of differentiation via upregulation of autophagy. However, in comparison with immortalized keratinocytes HaCaT, GA is more effective in inducing differentiation of carcinoma A431 cells, probably due to the inherently lower differentiation status of A431 cells. The activation of differentiation in poorly differentiated and highly malignant A431 cells by GA may lower the level of malignancy of these cells and decrease their tumorigenic potential.

## 1. Introduction

Plant hormones are biologically active substances of low molecular weight and diverse chemical composition. They are produced by plant cells and regulate many physiological processes, such as stress responses, stimulation or inhibition of plant development, growth, and differentiation [1]. Some plant hormones and/or their derivatives are approved and well-known drugs (salicylates) or bioactive compounds, which are used in cosmeceutical products (jasmonates) [2,3]. As components of natural origin with specific activity, plant hormones have been appealing objects of investigation in terms of their impact on animal and human cells. It has been demonstrated that certain plant hormones can affect specific cellular functions and even exhibit selective cytotoxicity against tumor cells [4,5,6,7], and molecular modifications of these compounds might substantially enhance these properties [3,8].

The gibberellin family of plant hormones includes structurally related tetracyclic diterpenes, which are produced by plants and fungi [9,10] and play an important role in main physiological processes, such as the stimulation of seeds germination; the growth of roots, stems, and leaves; and the development of flowers and fruits [11,12,13]. As a potent plant growth regulator, gibberellic acid (gibberellin A3) (GA) is often used in agriculture to activate plant growth and improve productivity. For instance, GA is applied to activate seeds after the period of dormancy, stimulate flowering, increase fruit size, and promote the development of seedless fruits and berries. Eventually, GA enters the food chain of animals and humans through regular consumption of plant foods and due to the commercial use of GA-containing products. As with many other biologically active plant-related compounds, excessive consumption of GA may have neutral, beneficial, or hazardous effects on animal and human health.

Despite that, our knowledge about the potential influence of GA on animals, and especially humans, is surprisingly limited. One of the first studies, published in 1988, showed that prolonged administration of GA (twice a week for 5 months) to Egyptian toads (Bufo regularis) induced the formation of hepatocellular carcinomas in 16% of experimental animals [14]. The carcinogenic effect of GA administration for 22 months was confirmed in Swiss albino mice. The study showed that 18% of the males and 36% of the females developed sebaceous adenomas, breast, and lung adenocarcinomas [15]. In Wistar albino rats, sub-chronic exposure to GA increased inflammatory skin and bladder disease via the activation and recruitment of mast cells [16]. The hepatotoxic effect of GA was also demonstrated in female rats and their offspring: experimental animals developed liver damage, had elevated plasma aminotransferases, bilirubin, and albumin levels, and increased lactate dehydrogenase activity along with other pathological changes in blood parameters [17,18].

However, more recent work showed that GA and its derivatives had a diverse but rather positive effect on cultured human cells. For instance, GA induced the production of zinc finger protein A (negative regulator of NF-κB-driven inflammation), which attenuated the inflammation in human primary nasal epithelial cells and a bronchial epithelial cell line (16HBE14o-) [19]. GA also stimulated the synthesis of α-amylase in adipose tissue-derived mesenchymal stem cells [20]. Remarkably, both research groups [19,20] did not report a cytotoxic effect of GA on cultured human cells of epithelial and mesenchymal origin. In addition, synthetic derivatives of gibberellins have demonstrated potent anti-cancerogenic and anti-angiogenic activity both in vitro and in vivo [8,21,22].

We summarized the experimental evidence obtained over the past years and concluded that the effect of GA on animals and humans is still far from clear. In vivo, GA exhibited a rather negative effect and might be viewed as a hazardous agent. However, when studied in vitro, GA and its derivatives had a rather positive influence on cellular functions. These discrepancies might be explained by the different experimental approaches, schemes of drug application, concentrations, and duration of exposure to GA. Evidently, only using standardized and comparable cellular models can help to elucidate the cellular response to GA treatment and follow the implications of this response.

One of our first goals was to investigate whether GA exerts the same effect on normal and pathologically changed cells of the same tissue origin. In our previous work, we analyzed the response of cultured human cells of epidermoid origin (immortalized non-tumorigenic keratinocytes HaCaT and carcinoma A431 cells) to GA and found that GA induced swelling and expansion of the Golgi complex in both cell lines [23]. The enlargement of the compartments of the biosynthetic system is often attributed to the development of ER stress, which is a major cellular response triggered upon excessive accumulation of misfolded proteins in the ER lumen [24,25]. Subsequently, cells activate UPR (unfolded protein response) to restore ER homeostasis. If the stress conditions are mild, this may lead to metabolic adaptation and survival of the cells, and in case of severe damage, UPR leads to the activation of cell death [26,27]. One of the potential strategies of cell survival may be the upregulation of autophagy [28] and activation of differentiation, specifically in the cells of epidermoid origin [29,30].

Thus, the investigation of the underlying effects of GA on animal and human cell lines may be important to address the discrepancy in GA action when tested in vitro and in vivo. Our previous study showed that in human cell lines, HaCaT and A431 GA induced the enlargement and swelling of the Golgi complex, which is a key compartment of the biosynthetic system [23]. We hypothesized these structural alterations could be a manifestation or a consequence of ER stress. Hence, we investigated the potential ability of non-toxic concentrations of GA to activate genes involved in the ER stress response and followed its effects in cultured human cells—immortalized non-tumorigenic keratinocytes (HaCaT) and epidermoid carcinoma cell line A431.

## 2. Materials and Methods

### 2.1. Reagents

GA3 (G7645) (Sigma-Aldrich, Saint-Quentin-Fallavier, France) diluted with 96° ethanol and stored at −6 °C as the stock solution; dithiothreitol (DTT) (A1101) (Appli-Chem, Darmstadt, Germany) diluted with cell medium and was used once; ER-Tracker Red (BODIPY™ TR Glibenclamide) (E34250) (Thermo Fisher Scientific, Bucharest, Romania, Waltham, MA, USA); rabbit polyclonal antibodies specific to GRP78 (G8918) (Sigma-Aldrich, St. Louis, MI, USA), (PA5-22967) (Thermo Fisher Scientific); LC3B (ab51520) (Abcam, Cambridge, UK), filaggrin (ab81468) (Abcam); mouse monoclonal antibodies specific to involucrin (I9018) (Sigma-Aldrich); Alexa Fluor-488-conjugated rabbit anti-IgG antibodies (ab150077) (Abcam); Alexa Fluor-488-conjugated mouse anti-IgG antibodies (A-11001) (Thermo Fisher Scientific).

### 2.2. Cell Cultures

HaCaT (immortalized non-tumorigenic human keratinocytes) (Table S1), A431 (human epidermoid carcinoma cells), human keratinocytes (passage 2 and passage 3), and HeLa (human adenocarcinoma cells) were kindly provided by the Cell Culture Collection for Biotechnological and Biomedical Research, Koltsov Institute of Developmental Biology, Russian Academy of Sciences. The cells were grown in DMEM (Dulbecco Modified Eagle’s Medium; PanEco, Moscow, Russia) (HaCaT and A431) or DMEM/F-12 (human keratinocytes and HeLa) supplemented with 10% fetal bovine serum (FBS) (HyClone, Logan, UT, USA), 2 mM L-glutamine (PanEco), 80 μg/mL gentamicin (Belmedpreparaty, Minsk, Belarus), and 0.2% Defined Keratinocyte-SFM Growth Supplement (Thermo Fisher Scientific) (only for human keratinocytes) under standard conditions (37 °C, 5% CO_2_). The cells were collected from the surface of a plastic flask with a 1:3 mixture of trypsin solution (PanEco) and Versene (0.2% EDTA in phosphate buffer) (PanEco) and plated on glass cover slips or on Petri dishes with glass bottom and grown for 48 h. Then, GA or DTT was added, and cells were incubated for 24 h.

### 2.3. Real-Time qPCR

RT qPCR was performed as described by Potashnikova et al. [31] using the following primers (Sintol, Moscow, Russia): for the *CHOP* gene, 5′-AGTCTAAGGCACTGAGCGTATC-3′/5′-TCTGTTTCCGTTTCCTGGTT-3′ [32]; for the *GRP78* gene, 5′-TCTGCTTGATGTGTGTCCTCTT-3′/5′-GTCGTTCACCTTCGTAGACCT-3′ [33]; for the *ATF4* gene, 5′-TGGCTGGCTGTGGATGG-3′/5′-TCCCGGAGAAGGCATCCT-3′ [34]; for the spliced form of mRNA of *XBP1* (X-box binding protein 1) gene, 5′-GCTGAGTCCGCAGCAGGT-3′/5′-CAGGGTCCAACTTGAACAGAAT-3′ (modification of the original sequence from [35]). The following primer sequences were used to amplify reference genes [36]: for the *ubiquitin* gene, 5′-ATTTGGGTCGCGGTTCTTG-3′/5′-TGCCTTGACATTCTCGATGGT-3′; for the *HPRT* gene, 5′-TGACACTGGCAAAACAATGCA-3′/5′-GGTCCTTTTCACCAGCAAGCT-3′; for the *GAPDH* gene, 5′-TGCACCACAACTGCTTAGC-3′/5′- GGCATGGACTGTGGTCATGAG-3′; for the *YWHAZ* gene, 5′-ACTTTTGGTACATTGTGGCTTCAA-3′/5′–CCGCCAGGACAAACCAGTAT-3′. All samples were processed in triplicate. One sample of cDNA put into each PCR run served as an inter-run calibrator for uniting data into one experiment. Primer specificity was confirmed by melting curve analysis and detection of products with predicted length using 1.5% agarose gel electrophoresis. Amplification efficiency (E) was calculated as E = [10(−1/slope) − 1], using the slope of the semi-log regression plot of Cq versus log input of cDNA. Each reaction was performed in triplicate. Results were analyzed according to Vandesompele et al. [36].

### 2.4. MTT Assay

The viability of cells in the presence of different concentrations of GA was estimated using an MTT assay performed as described earlier [7,23].

### 2.5. Live Fluorescent Staining and Immunocytochemistry

For ER-tracker staining, 1 µL of 1 mM ER-tracker solution was added to 1 mL cell culture medium for 30 min at 37 °C. After staining, the cells were washed with medium for 5 min at 37 °C and visualized under a fluorescence microscope.

For immunocytochemical staining, the cells were fixed with 4% formaldehyde (MP Biochemical, Illkirch-Graffenstaden, France) in PBS (pH 7.2–7.4) and treated with 0.1% Triton X-100 (Serva, Heidelberg, Germany). For anti-filaggrin staining, the cells were fixed with methanol (Merck, Darmstadt, Germany). Nuclei were visualized with DAPI (4′,6-diamidine2′-phenylindole dihydrochloride; Sigma-Aldrich), and the preparations were embedded in Mowiol (Hoechst, Klipphausen, Germany).

All preparations were analyzed under an Axiovert 200M inverted fluorescence microscope (Carl Zeiss Inc., Oberkochen, Germany; PlanApo 20× and 63×/1.4 oil objectives) equipped with Carl Zeiss (Jena, Gemany) AxioCam black-and-white digital camera with AxioVision 3.1 (Carl Zeiss, Jena, Germany) software.

### 2.6. Transmission Electron Microscopy (TEM)

The cells were fixed for 30 min with 2.5% glutaraldehyde (Ted Pella Inc., Redding, CA, USA), washed with PBS (pH 7.2–7.4), and postfixed with 1% OsO4 solution in PBS (pH 7.2–7.4; Sigma-Aldrich) for 1 h. Dehydration and embedding in Epon 812 (Sigma-Aldrich) were performed using the standard technique. Ultrathin sections of Epon-embedded samples were stained with 1.5% uranyl acetate solution and lead citrate according to Reynolds [37] and analyzed with a JEM-1011 transmission electron microscope (JEOL, Tokyo, Japan) equipped with a GATAN ES500W digital camera operated by the Digital Micrograph GATAN software.

### 2.7. Western Blot Analysis

The cells were lysed in 2× lysis buffer containing 200 mM Tris-HCl, protease inhibitor cocktail (Roche, Pleasanton, CA, USA), 400 mM β-mercaptoethanol (Bio-Rad Laboratories, Inc., Hercules, CA, USA), 4% sodium dodecyl sulfate (SDS; Serva), 0.01% bromophenol blue, and 40% glycerol (PanReac, Barcelona, Spain) and incubated at 95 °C for 1 min. Proteins were separated using 10% or 12.5% sodium dodecyl sulfate polyacrylamide gel electrophoresis (SDS-PAGE) and transferred from the gel to PVDF membranes by a semi-wet approach using Trans-Blot^®^ Turbo™ RTA Mini LF PVDF TransferKit (Bio-Rad Laboratories, Inc.). Membranes were blocked with 5% milk on tris-buffered saline containing 0.1% Tween (TTBS) for 1 h at room temperature, then stained overnight with primary antibodies against GAPDH, GRP78, LC3B, involucrin, filaggrin, and subsequently with HRP-conjugated secondary antibodies (Bio-Rad Laboratories, Inc., Hercules, CA, USA). Target proteins were visualized by Novex ECL Kit (Invitrogen, Waltham, MA, USA) in the ChemiDoc™ visualization system (Bio-Rad Laboratories, Inc.). Optical density of the protein bands was determined using ImageLab Software (Bio-Rad Laboratories, Inc.).

### 2.8. Data Analysis

The images were processed with ImageJ software (National Institutes of Health, Bathesda, MD, USA) and GIMP 2.8.18 free software. Statistical data analysis was performed in GraphPad Prizm 6 (GraphPad Software, San Diego, CA, USA) and using the Mann–Whitney U-test (nonparametric); the differences were considered statistically significant at *p* < 0.05.

## 3. Results

### 3.1. The Effect of GA on Metabolic Activity of Cells

Beforehand, we analyzed the data of other authors on the effect of plant hormones on animal and human cells [38]. We found that in most experiments, hormones acted at micromolar and even millimolar concentrations, combined with prolonged time of treatment. For instance, Kasamatsu et al. [20] demonstrated that GA did not affect the viability of adipose-derived stem cells in a dose- (up to 1mM) or time-dependent manner. Using MTT assay, we evaluated the metabolic activity (cell viability) of HaCaT and A431 cells, growing for 15, 24, 48, 72, and 96 h in the presence of GA and at concentrations varying from 0.5 to 4 mM (Figure S1). First, we compared the optical density of the samples growing without (C1) and with ethanol as a solvent for GA (C2) and did not observe a significant difference between C1 and C2; therefore, we used C2 as a reference sample. The incubation of HaCaT and A431 cell with 0.5–4 mM of GA up to 96 h did not influence the cell viability (Figure S1a,b). Nonetheless, a small but statistically significant difference in reduction of metabolic activity was detected after incubation of both cell lines with 1, 2, and 4 mM of GA for 24 h. These results showed that GA did not affect the viability of HaCaT and A431 cells in a time- and dose-dependent manner. To confirm that GA does not have a cytotoxic effect on the other cells of epidermoid origin, we incubated human keratinocytes and HeLa cells in the same experimental conditions as HaCaT and A431 cells. The results demonstrated that GA also did not affect the metabolic activity of these cells (Figure S2). In our previous study, we showed that GA (2 mM for 24 h) induced the swelling and expansion of the Golgi complex in HaCaT and A431 cells [23]; therefore, we used this dose and timing for further experiments.

### 3.2. GA Induces Upregulation of ER Stress Genes

**Figure 1:**
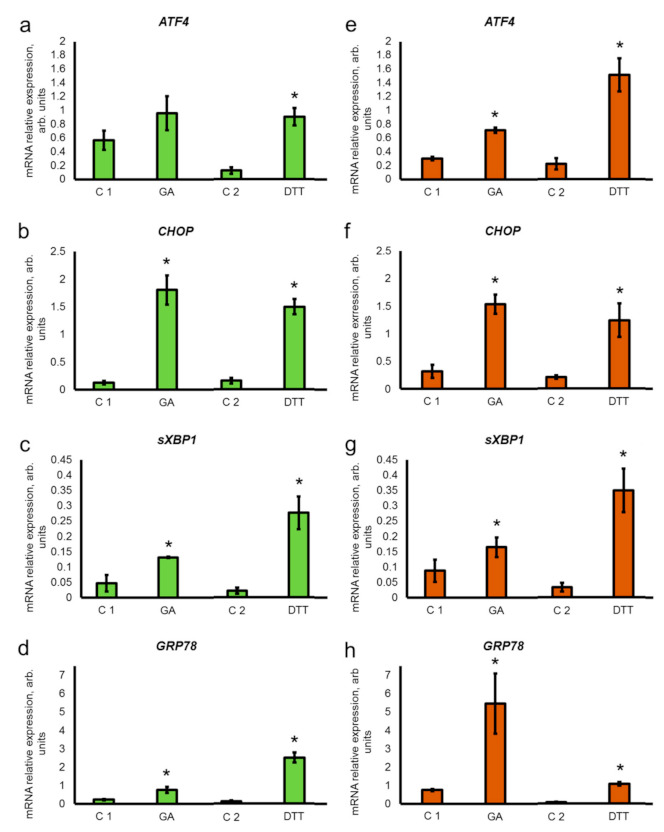
Levels of mRNA expression of the ER stress genes *ATF4*, *CHOP*, *sXBP1*, and *GRP78* in keratinocytes HaCaT (**a**–**d**) and carcinoma A431 cells (**e**–**h**) after 24 h of incubation with 2 mM of gibberellic acid (GA) and 2 mM dithiothreitol (DTT). C1—control for GA when ethanol as a solvent for GA is added to the cultivation medium; C2—control for DTT when cultivation medium as a solvent for DTT is added to the cultivation medium. Data are shown as mean ± average error (from 3 replicates of 3–4 independent experiments); * *p* ≤ 0.05 according to the Mann–Whitney test.

Using RT-qPCR, we investigated the effect of GA on the expression of genes involved in the ER stress response—*ATF4* (activating transcription factor 4), *CHOP* (C/EBP homologous protein), spliced form of XBP1 (X-box binding protein 1) and *GRP78* (binding immunoglobulin protein (BiP)/78-kDa glucose-regulated protein). The ER stress activator dithiothreitol (DTT) was used as a positive control, and as expected, it induced up-regulation of ER stress genes in both cell lines (Figure 1a–h).

We found that GA upregulated the expression of ER stress genes in both cell lines. As shown in Figure 1, in HaCaT cells, the level of expression of *CHOP* was elevated 14.8-fold (Figure 1b), for *sXBP1* transcript 2.8-fold (Figure 1c) and 3.1-fold for *GRP78* (Figure 1d). In A431 cells, GA upregulated the expression of *ATF4* 2.3-fold (Figure 1e); CHOP—4.8-fold (Figure 1f); *sXBP1* transcript—1.9-fold (Figure 1g); and *GRP78*—7.2-fold (Figure 1h). However, the difference between the transcriptional level of *ATF4* in the control sample (C2) and in the presence of GA was not statistically significant in HaCaT cells (Figure 1a), suggesting that under these experimental conditions, only a trend towards *ATF4* up-regulation was detected.

### 3.3. Detection of ER Stress Regulator GRP78

First, we studied the cellular distribution of the ER using live staining with ER-Tracker Red (Figure S3a–h). In both cell lines, the ER appeared as a tubular network extending from the perinuclear cytoplasm towards the cell periphery (Figure S3a,b,e,f), and neither the distribution nor the morphology of the network was affected by GA treatment (Figure S3c,d,g,h). Concomitantly, we investigated the ultrastructure of the ER cisternae in both cell lines (Figure S3i–m) and did not detect swelling or any other structural ER alterations in the presence of GA. These results confirm that ER stress during GA treatment, which was detected by RT-qPCR, is not accompanied by noticeable changes in the ER distribution and structure.

**Figure 2:**
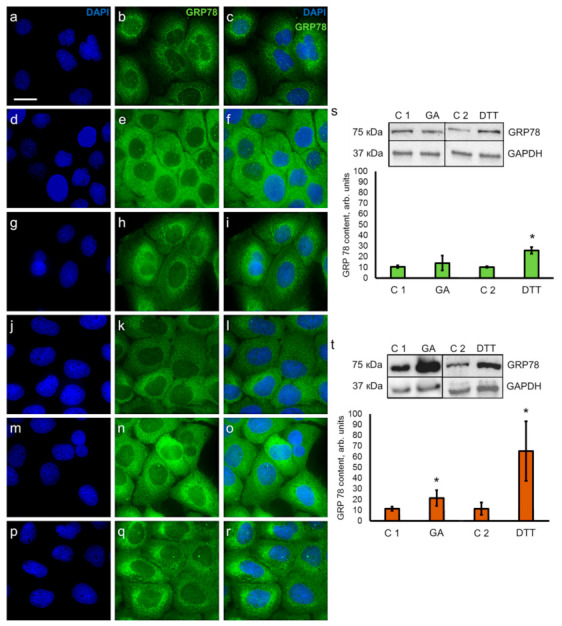
Immunocytochemical visualization of GRP78 localization and analysis of GRP78 content in keratinocytes HaCaT and carcinoma A431 cells. (**a**–**i**) Keratinocytes HaCaT in control specimens (**a**–**c**) and in the presence of DTT (**d**–**f**) and GA (**g**,**h**); (**j**–**r**) carcinoma A431 cells in control specimens (**j**–**l**), in the presence of DTT (**m**–**o**) and GA (**p**–**r**). Nuclei are stained with DAPI (left column); GRP78 is visualized with anti-GRP78 antibodies (middle column), merged images (right column). Scale bar, 20 μm. Western blot analysis and evaluation of GRP78 content in HaCaT (**s**) and A431 cells (**t**). C1—cells growing with ethanol as a solvent for GA (control for GA); C2—cells growing in standard conditions (control for DTT). Data are shown as mean ± standard deviation (*n* = 3–5); * *p* ≤ 0.05 according to the Mann–Whitney test.

Next, we studied the localization of GRP78 in control HaCaT and A431 cells (Figure 2a–c,j–l), DTT-treated cells (Figure 2d–f,m–o), and GA-treated cells (Figure 2g–i,p–r). Immunocytochemical visualization of GRP78 showed that it labeled the tubular network in both cell lines, which was mainly accumulated around the nucleus. The pattern of GRP78 labelling was comparable to the pattern of staining obtained using ER-tracker Red shown in Figure S2. Additionally, more bright diffuse staining of the cytoplasm was observed in both cell lines in the presence of DTT (Figure 2d–f,m–o) and in A431 cells after GA treatment (Figure 2p–r). The intensity of staining may reflect the increased production of GRP78; therefore, we analyzed the content of GRP78 in HaCaT (Figure 2s) and A431 cells (Figure 2t) using Western blot using DTT as a positive control for GRP78.

We found that GA induced statistically validated elevation of GRP78 content (1.9 times) only in A431 cells (Figure 2t), but a trend towards the increase in GRP78 production was also observed in HaCaT cells (Figure 2s). These results confirm the difference in the intensity of GRP78 immunostaining seen in Figure 2a–r. As mentioned in Section 3.2, GA induced the upregulation of GRP78 genes in both HaCaT and A431 cell lines, yet in HaCaT cells, the upregulation of GRP78 expression was not as pronounced as in A431 cells (Figure 1d,i). This difference in activation of GRP78 genes by GA may explain the lower level of GRP78 synthesis seen in HaCaT cells, which was detected by Western blot analysis (Figure 2s).

### 3.4. GA increases the Content of Autophagy Marker LC3-II

**Figure 3:**
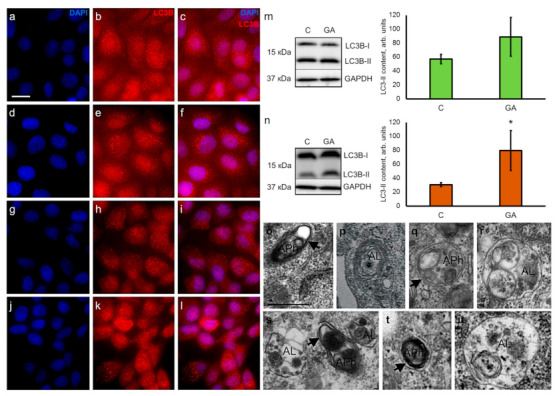
Immunocytochemical visualization of autophagy marker LC3B and analysis of LC3B-II content in keratinocytes HaCaT and carcinoma A431 cells. (**a**–**f**) Keratinocytes HaCaT in control specimens (**a**–**c**) and in the presence of GA (**d**–**f**); (**g**–**l**) carcinoma A431 cells in control specimens (**g**–**i**) and in the presence of GA (**j**–**l**). Nuclei are stained with DAPI (left column), autophagosomes are visualized with anti-LC3B antibodies (middle column), merged images (right column). Scale bar, 20 μm. Western blot analysis and evaluation of LC3B-II content in HaCaT (**m**) and A431 (**n**) cells. C—cells growing with ethanol as a solvent for GA (control for GA). Data are shown as mean ± standard deviation (*n* = 3); * *p* ≤ 0.05 according to the Mann–Whitney test. TEM images of autophagosomes (APh) and late autolysosomes (AL) in HaCaT (**o**–**r**) and A431 cells (**s**–**u**). Autophagosomes and autolysosomes are present in control specimens of HaCaT (**o**,**p**) and A431 cells (**s**), and in GA-treated HaCaT (**q**,**r**) and A431 cells (**t**,**u**). Arrowheads point to the gap between two membranes surrounding autophagosomes. Scale bar, 0.5 μm.

It is well known that ER stress is a trigger for autophagy, which may cause reparative autophagy or autophagic cell death [39,40,41]. We analyzed the presence of LC3B as the autophagy marker in control and GA-treated cells. Immunocytochemical visualization of autophagosomes labeled with antibodies against LC3B did not show detectable changes in the pattern of LC3B staining in both cell lines before and after GA treatment (Figure 3a–l). However, Western blot analysis demonstrated an increased level of LC3B-II content in GA-treated HaCaT cells (Figure 3m) and A431 cells (Figure 3n), but the difference was only significant for A431 cells. We believe that similar to GRP78, HaCaT cells display a tendency for LC3B-II production. The presence of autophagosomes and autolysosomes, typical for different stages of constitutive and induced autophagy, was confirmed by TEM analysis in HaCaT and A431 cells (Figure 3o–u).

### 3.5. GA Raises the Level of Epidermal Differentiation Markers Involucrin and Filaggrin

It has been shown that ER stress and autophagy play an important role in the differentiation of human keratinocytes [42]. Therefore, we investigated the levels of epidermal differentiation markers—involucrin and filaggrin—in both cell lines in the presence of GA.

**Figure 4:**
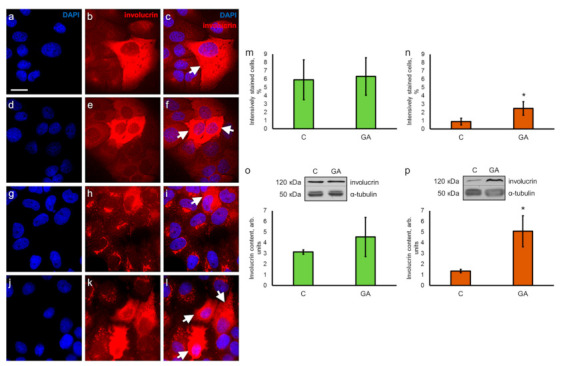
Immunocytochemical visualization and evaluation of involucrin content in keratinocytes HaCaT and carcinoma A431 cells. (**a**–**f**) Keratinocytes HaCaT in control specimens (**a**–**c**) and in the presence of GA (**d**–**f**), arrowheads indicate brightly stained cells; (**g**–**l**) carcinoma A431 cells in control specimens (**g**–**i**) and in the presence of GA (**j**–**l**); arrowheads indicate cells with bright diffuse staining of the cytoplasm; note that involucrin-positive tubular/vesicular structures are seen in the cells with faintly stained cytoplasm. Nuclei are stained with DAPI (left column), involucrin-positive staining is detected with anti-involucrin antibodies (middle column), merged images (right column). Scale bar, 20 μm. The relative number of cells with brightly stained cytoplasm in control specimens versus GA treated samples is shown in (**m**) (HaCaT cells) and (**n**) (A431 cells). Data are shown as mean ± standard deviation (*n* = 3); * *p* ≤ 0.01 according to the Mann–Whitney test. Western blot analysis and evaluation of involucrin content in HaCaT (**o**) and A431 cells (**p**). C—cells growing with ethanol as a solvent for GA (control for GA). Data are shown as mean ± standard deviation (*n* = 3); * *p* ≤ 0.05 according to the Mann–Whitney test.

Immunocytochemical staining of HaCaT cells with antibodies against involucrin showed that the intensity of cytoplasmic staining varied across different cells (Figure 4a–c). The brightly stained cells (5.9% of the whole population, Figure 4m, C) were either singular or assembled into clusters. Staining with DAPI confirmed that these cells were not debris or remnants of dead cells (Figure 4c). The same pattern of staining was observed after incubation with GA (Figure 4d–f), and the number of brightly stained cells did not change (6.3% of the whole population, Figure 4m, GA).

However, we detected a completely different pattern of staining in A431 cells. Most of the cells had an involucrin-positive tubular/vesicular network distributed next to or around the nucleus and arranged similar to the Golgi complex (Figure 4g–i). The bright diffuse staining of the cytoplasm (Figure 4i, white arrowhead) was observed in only 0.9% of cells (Figure 4n, C). In GA-treated cells, the number of cells with prominent diffuse staining of the cytoplasm (Figure 4l, white arrowheads) was increased up to 2.5% (Figure 4n, GA). These observations were supported by the Western blot analysis; it demonstrated that GA increased the involucrin content 3.8 times in A431 cells (Figure 4p), and once again, HaCaT cells showed the tendency for the elevation of involucrin production (Figure 4o). Notably, in A431 cells growing in the presence of GA, involucrin-positive tubular/vesicular structures changed their localization and expanded from the perinuclear region towards the cell periphery (Figure S4). In our previous study, we reported comparable redistribution of Golgi complex in GA-treated A431 cells [23].

Immunocytochemical staining for filaggrin showed diffuse or dot-loke staining of the cytoplasm in HaCaT cells (Figure S5a–c), and the same pattern of staining was observed during GA treatment (Figure S5d–f). Reticulated and dot-like pattern of staining for filaggrin was also observed in A431 cells (Figure S5g–i), and this pattern was not changed during GA treatment either (Figure S5j–l). However, Western blot analysis showed that GA increased the content of filaggrin 2.5-fold in both cell lines (Figure S5m,n).

## 4. Discussion

Many external (physical and environmental conditions, chemical agents, viral infections) and internal factors (gene mutations, depletion of Ca^2+^ levels) may induce the accumulation of unfolded or misfolded proteins in the ER lumen and activate the development of the ER stress. Adaptive cellular reaction termed unfolded protein response (UPR) restores ER homeostasis via the activation of signaling mechanisms, which are transduced by transmembrane ER-resident sensors/receptors—IRE1α (inositol-requiring protein 1α), ATF6 (activating transcription factor 6), and PERK (protein kinase RNA-like endoplasmic reticulum kinase) [43,44,45]. Normally, the association with BIP/GRP78, which is predominantly localized in the ER lumen, inactivates these sensors. Upon UPR activation, GRP78 dissociates from PERK, ATF6, and IRE1α, and released sensors activate downstream signaling pathways, which either result in cellular death or survival [26,46,47].

We found that plant hormone GA increased the expressional level of mRNA for the ER stress genes (*ATF4*, *CHOP*, *sXBP1*, and *GRP78*) in cultured human cells of epidermoid origin—immortalized keratinocytes HaCaT and carcinoma A431cells—but in a slightly different manner for *ATF4*. The level of mRNA for *CHOP*, *sXBP1*, and *GRP78* increased in both cell lines, but in HaCaT cells, only a tendency for the upregulation of *ATF4* expression was seen. During mild ER stress and short UPR, eukaryotic cells utilize several compensatory mechanisms which aim to reduce the stress and restore ER homeostasis [27,28,40,48]. Strong ER stress and/or prolonged UPR may halt these compensatory reactions and trigger the initiation of cell death [27,30,44]. We did not detect the decrease of cell viability in the presence of GA and presumed that upregulation of *ATF4*, *CHOP*, *sXBP1*, and *GRP78* is a manifestation of mild ER stress in HaCaT and A431 cells.

ATF4 and CHOP are members of the PERK signaling mechanism. After dissociation from GRP78, PERK completely blocks the translation and phosphorylates eIF2α (eukaryotic initiation factor 2α). This, in turn, activates the translation of ATF4 and the development of adaptive response. At the same time, ATF4 may activate CHOP signaling pathways and facilitate the initiation of apoptosis [30,49,50]. XBP1 is a component of the most conservative IRE1α signaling pathway in eukaryotes and the main regulator of the adaptation to ER stress [51]. After the activation of the ER stress response, the endoribonuclease domain of IRE1α initiates splicing of XBP1 mRNA [52], and the spliced form of XBP1 mRNA is used for the synthesis of sXBP1, which is a major transcriptional factor for the genes involved in UPR and ERAD [51,53].

Unequivocally, increased expression of GRP78 is the ultimate feature of the ER stress [50]. We found that GA upregulated the expression of mRNA for GRP78 in both cell lines. However, the evaluation of the protein content using Western blotting only showed the increase in GRP78 production in A431 cells. It is likely that, under the current experimental conditions, the level of upregulation for *GRP78* expression in HaCaT is not sufficient to trigger the increased production of GRP78 protein. Thus, GA induced mild ER stress in HaCaT and A431 cells, but A431 cells were more “sensitive” to GA treatment as they had higher levels of upregulation for *ATF4* and *GRP78* genes and increased content of GRP78. Our findings are confirmed by recent studies about the effect of GA derivative—GA-13315 (13-chlorine-3,15-dioxy-gibberellic acid methyl ester) on cultured lung adenocarcinoma cells A549. This synthetic compound substantially increased the expression of GRP78, ATF4, and CHOP in A549 cells, but unlike GA, GA-13315 triggered apoptotic cell death [22].

It is known that ER stress may stimulate autophagy, and the latter is one of the mechanisms for the destruction of aggregated and misfolded proteins during ERAD [28,46]. We found that GA increased the LC3B-II content in A431 cells, yet only the tendency for elevated LC3-II production was detected in HaCaT cells. The increased level of LC3B-II production in A431 cells might be one of the consequences of the ER stress, which was also confirmed by the upregulation of *ATF4* expression and the increased level of GRP78 content. Notably, the same effect was observed in HaCaT cells, but to a lesser extent. PERK mediates the upregulation of LC3 and ATG5 via ATF4 and CHOP pathways in many cultured cell lines, thereby facilitating phagophore formation [54,55,56]. The activation of the IRE1/XBP1 signaling mechanism may also link UPR and autophagy, as shown by Kishino et al. [51]. Moreover, tumor cells may activate ATF4-mediated autophagy as a survival mechanism under stress conditions [57,58,59]. GRP78 also improves the survival of tumor cells during stress conditions because it stimulates macroautophagy and hampers proapoptotic signaling mechanisms [45,60].

We used TEM to investigate membranous vesicles and identified autophagosomes and autolysosomes in control and GA treated cells. Numerous autophagosomes and autolysosomes in control samples confirmed a high basal level of autophagy in both cell lines. However, the increased content of LC3B-II and the presence of autolysosomes in GA-treated A431 cells demonstrated the progression of autophagic flux without arrest at early stages. Moreover, the upregulation of *ATF4* expression and elevation of GRP78 content also confirmed the increase in autophagy in A431 cells exposed to GA.

Activation of autophagy promotes tumor progression and helps tumor cells to survive in stress conditions [61]. At the same time, autophagy plays a crucial role in normal physiological processes, including differentiation. For instance, autophagy triggers the early stages of signaling mechanisms that control the differentiation of immortalized keratinocytes HaCaT [42]. The differentiation is also one of the adaptive survival mechanisms in response to various external stimuli. It has been demonstrated that ER stress affects the biogenesis of lysosomes and the differentiation of primary neonatal human epidermal keratinocytes [29]. Terminal differentiation of murine epidermal keratinocytes is also accompanied by the activation of autophagy markers and nucleophagy [62]. In our experiments, GA mainly stimulated the production of LC3B-II in carcinoma cell line A431, yet the tendency for an elevated level of LC3B-II was also observed in HaCaT cells, suggesting that autophagy may further progress into a cellular reaction to stress and lead to the activation of differentiation.

Immortalized non-tumorigenic human keratinocytes HaCaT constitutively express epidermal differentiation markers (keratin 1, 10, involucrin, filaggrin) and form structurally organized and differentiated epidermal tissue after transplantation to naked mice [63]. During cultivation in vitro and in the presence of low concentration of retinoic acid, these cells may form the stratum corneum [64]. Derived from vulvar carcinoma, A431 cells are not able to differentiate even in favorable growth conditions (cultivation in serum-free medium, low concentration of retinoic acid, high concentration of Ca^2+^), but they can still produce involucrin [65]. Assessment of the autophagy marker proteins combined with proteins from the epidermal differentiation cluster (involucrin and filaggrin) is often used for the evaluation of the differentiation status of normal and tumor cells. We compared the levels of epidermal differentiation markers in both cell lines, cultivated in standard conditions and in the presence of GA, and found that GA stimulated the synthesis of involucrin in A431 cells and filaggrin—in HaCaT and A431 cells.

The enzymes from the transglutaminase family (predominantly transglutaminase I) catalyze the attachment of involucrin to the plasma membrane proteins [65,66]. However, the activity of transglutaminase I is suppressed in A431 cells [67]. This may explain the difference in the pattern of immunocytochemical staining for involucrin in HaCaT (Figure 4a–c) and A431 cells (Figure 4g–i). Only single A431 cells demonstrate the diffuse staining of the cytoplasm, and most cells have involucrin-positive tubular/vesicular structures, which have the appearance of the Golgi complex. We assume that the atypical pattern of involucrin staining in A431 cells may be attributed to the anomalous phenotype of the A431 cells. The level of involucrin production and the number of A431 cells with a normalized pattern of staining increased in the presence of GA. This demonstrates that GA may restore at least some features of normal phenotype in A431 cells.

It has been shown that filaggrin may be expressed in subpopulations of oral squamous carcinomas [68] and in cultivated squamous cell carcinoma (SCC-13) [69]. We detected filaggrin in HaCaT and A431 cells using immunocytochemical staining and Western blotting and showed that during incubation with GA, the level of filaggrin production increased in both cell lines.

It is known that keratinocyte differentiation within the epidermis depends upon mild ER stress, UPR [27], and consequent activation of the ATF4 pathway [70]. ATF4 also regulates differentiation in other cell types, including osteoblasts [71,72], adipocytes [73], chondrocytes [74], and breast epithelial cells [75]. The upregulation of ATF4 signaling and activation of differentiation markers (involucrin and filaggrin) primarily in A431 cells demonstrates that GA activates differentiation in A431 cells more effectively than in keratinocytes HaCaT.

Tumor cells often have a higher level of GRP78 (especially after therapy), which provides better conditions for their survival, active proliferation, increased drug resistance, and metastatic growth [45,76,77]. However, certain agents may induce upregulation of GRP78 expression and trigger rather positive changes in tumor cells by activating differentiation. This is crucial for highly malignant and undifferentiated types of tumors because activation of differentiation slows down or inhibits proliferation, changes cellular phenotype, and normalizes cellular functions [78,79]. These data are in agreement with our observation of up-regulation of GRP78 expression followed by increased production of GRP78 in carcinoma A431 cells exposed to GA. The increase of GRP78 expression is also observed during differentiation of normal cells, for instance, human keratinocytes [70], embryonic stem cells H9 [80], rat myofibroblasts [81], and human and murine lung fibroblasts [82].

Thus, we demonstrated that at non-toxic doses and duration of treatment, GA induced mild ER stress and autophagy followed by the activation of differentiation in cultured human cells of epidermoid origin—immortalized keratinocytes HaCaT and carcinoma A431 cells. It appeared that GA was more effective at activating differentiation in carcinoma A431 cells, probably due to the inherently lower differentiation status of these cells compared to HaCaT keratinocytes.

## 5. Conclusions

Plant hormone GA added to cultured human non-tumorigenic immortalized keratinocytes HaCaT and epidermoid carcinoma A431 cells does not produce a cytotoxic effect. However, it may induce mild ER stress, which stimulates cell differentiation via the activation and enhancement of autophagy. Overall, the effect of GA on cell differentiation was more pronounced in A431 cells than in HaCaT, which may be explained by the lower initial differentiation status of A431 cells. The activation of differentiation triggered by GA may be viewed as a positive effect in poorly differentiated and highly malignant cells, as the activation of at least some differentiation features may lower the level of malignancy of tumor cells and decrease their tumorigenic potential. Therefore, under these conditions, GA may be an anti-cancerogenic agent. Our study also demonstrates that GA induces the activation of differentiation in HaCaT cells, but to a lesser extent. Upregulation of differentiation may be positive for cells with functional deficiencies (ageing and senescent cells) and can have negative effects on cells with enhanced differentiation features, such as during hyperkeratosis. Further studies may provide insight into using GA and its derivatives for activation of cell differentiation and, potentially, other cellular processes.

## Data Availability

The data that support the findings presented in this manuscript are available from the corresponding author upon request.

## Supplementary Materials

The following are available online at www.mdpi.com/article/10.3390/pharmaceutics13111813/s1, Table S1: The genetic information for HaCaT cell line, Figure S1: Evaluation of metabolic activity of keratinocytes HaCaT (a) and carcinoma A431 cells (b) with MTT assay, Figure S2: Evaluation of metabolic activity of human keratinocytes (a) and adenocarcinoma HeLa cells (b) with MTT assay, Figure S3: Visualization of localization and ER structure in keratinocytes HaCaT and carcinoma A431 cells using light and electron microscopy, Figure S4: Immunocytochemical visualization of tubular/vesicular network with antibodies against involucrin in carcinoma A431 cells, Figure S5: Immunocytochemical visualization and evaluation of filaggrin content in keratinocytes HaCaT and carcinoma A431 cells.
