# Supplementary material for: Gibberellic Acid Initiates ER Stress and Activation of Differentiation in Cultured Human Immortalized Keratinocytes HaCaT and Epidermoid Carcinoma Cells A431
Source: Pharmaceutics. 2021 Oct 30;13(11):1813. doi: 10.3390/pharmaceutics13111813 (PMC8622727; doi:10.3390/pharmaceutics13111813)
Supplement: Supplementary file 1 [file pharmaceutics-13-01813-s001.zip › pharmaceutics-1410509 - corrected.pdf]

pharmaceutics

MDPI

Supplementary Materials: Gibberellic Acid Initiates ER Stress and Activation of Differentiation in Cultured Human Immortalized Keratinocytes HaCaT and Epidermoid Carcinoma Cells A431

Mariya Vildanova, Polina Vishnyakova, Aleena Saidova, Victoria Konduktorova, Galina Onishchenko and Elena Smirnova

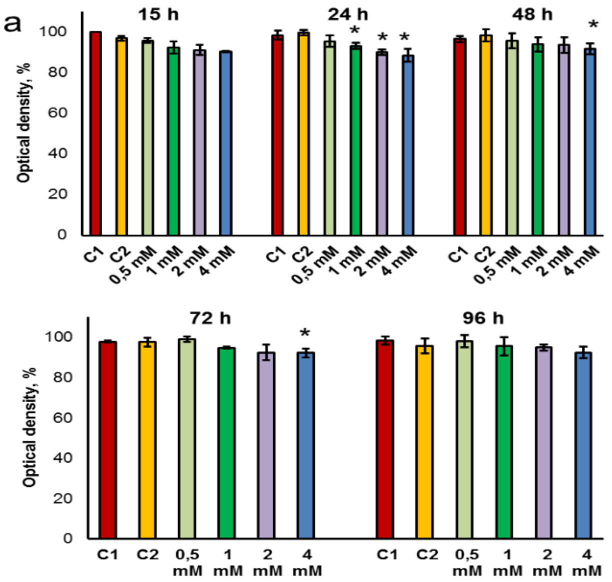

	C1	C2	0,5 mM	1 mM	2 mM	4 mM
15 h	100%	97%±1,2	95,9%± 1,1	92,5%± 2,87	91,3%± 2,38	90,45%± 0,34
	n=4	n=4	n=2 P=0,8	n=2 P=0,27	n=2 P=0,13	n=2 P=0,13
24 h	98,59%± 2,09	99,82%± 1,21	95,65%± 2,95	93,3%± 1,5	90,24%± 1,3	88,61%± 3,24
	n=5	n=6	n=5 P=0,017	n=5 P=0,004	n=4 P=0,01	n=4 P=0,01
48 h	96,77%± 1,53	98,49%± 2,96	95,9%± 3,62	94%± 3,44	93,76%± 3,81	91,83%± 2,68
	n=6	n=7	n=4	n=5	n=5	n=6 P=0,008
72 h	97,83%± 0,55	97,57%± 2,12	98,97%± 1,31	94,8%± 0,46	92,52%± 3,84	92,3%± 2,18
	n=3	n=3	n=3	n=3 P=0,1	n=4 P=0,1143	n=4 P=0,029
96 h	98,3%± 2,07	95,57%± 3,7	98%± 3,12	95,6%± 4,44	94,9%± 1,63	92,43%± 2,83
	n=3	n=3	n=3	n=3	n=4	n=4

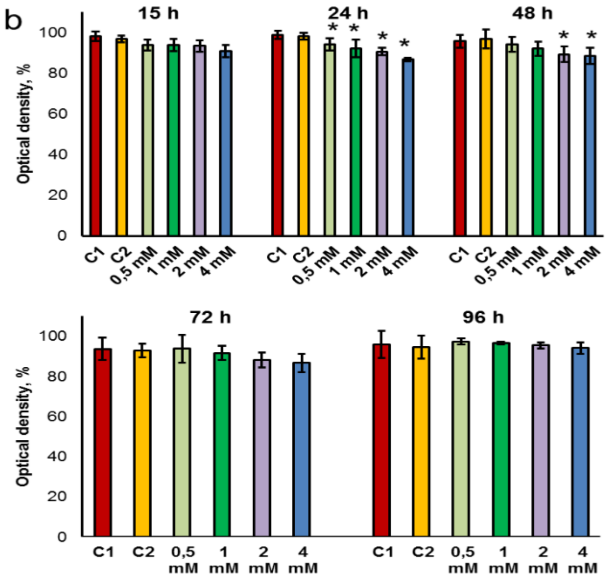

	C1	C2	0,5 mM	1 mM	2 mM	4 mM
15 h	98,14%± 2,43	96,88%± 1,76	93,87%± 2,58	93,83%± 3,05	93,46%± 2,8	90,85%± 3,05
	n=4	n=4	n=2 P=0,27	n=2 P=0,27	n=2 P=0,27	n=2 P=0,13
24 h	98,75%± 1,95	98,11%± 1,68	94,14%± 2,89	92,18%± 4,47	90,6%± 1,79	86,79%± 0,84
	n=6	n=6	n=5 P=0,03	n=4 P=0,01	n=4 P=0,01	n=4 P=0,01
48 h	95,76%± 3,2	99,8%± 4,72	94,28%± 3,68	92,06%± 3,48	89,32%± 3,96	88,54%± 3,95
	n=5	n=5	n=5	n=5	n=6 P=0,0498	n=6 P=0,028
72 h	93,73%± 5,6	92,97%± 3,4	93,83%± 6,95	91,73%± 3,67	88,2%± 3,8	86,83%± 4,58
	n=3	n=3	n=3	n=3	n=3	n=4 P=0,4
96 h	96,07%± 6,81	94,6%± 5,79	97,43%± 1,55	96,77%± 0,65	95,6%± 1,51	94,15%± 2,95
	n=3	n=3	n=3	n=3	n=4	n=3

Figure S1. Evaluation of metabolic activity of keratinocytes HaCaT (**a**) and carcinoma A431 cells (**b**) with MTT assay. Both cell lines are treated with 0.5–4 mM of GA and incubated for 15 h and 24 h. C1 — cells growing in standard conditions (control); C2 — cells growing with ethanol as a solvent

for GA (control for GA). The rate of metabolic activity after incubation with GA is compared with C2 samples. In each independent experiment (n), the maximal detected value of optical density is denoted as 100%. The results on metabolic activity rate, standard deviation, number of independent experiments (n) and P-values are also presented in corresponding tables. Data are shown as mean ± standard deviation (from 3–6 replicates of 2–6 independent experiments (n)); * $p \leq 0.05$ according to the Mann — Whitney test.

a

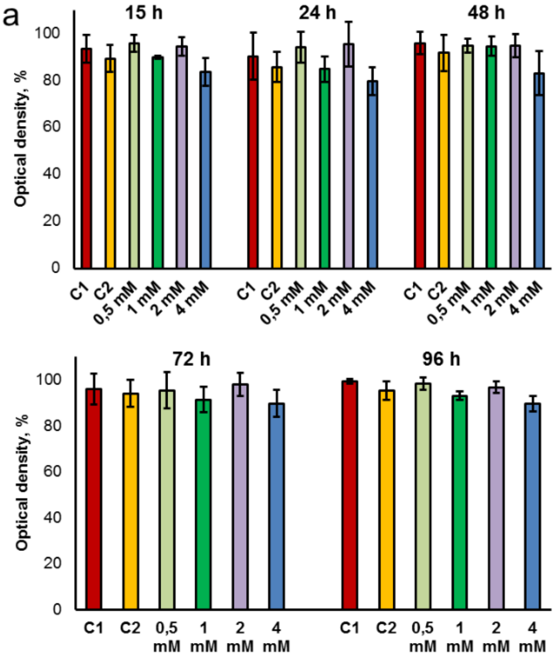

	C1	C2	0,5 mM	1 mM	2 mM	4 mM
15 h	93,7%± 5,9	89,47%± 5,77	95,97%± 3,54	90,2%± 0,65	94,67%± 3,8	83,83%± 5,77
	n=3	n=3	n=3	n=3	n=3	n=4 P=0,86
24 h	90%± 9,9	85,87%± 6,46	94,3%± 6,5	85,07%± 5,46	95,75%± 9,51	79,97%± 5,84
	n=3	n=3	n=3	n=3	n=3	n=4 P=0,26
48 h	96,1%± 4,69	91,93%± 7,65	94,97%± 2,89	94,7%± 4,13	95,15%± 4,95	83,33%± 9,37
	n=3	n=3	n=3	n=3	n=3	n=4
72 h	95,97%± 6,64	94,03%± 5,85	95,4%± 7,9	91,43%± 5,54	98,09%± 5,07	89,77%± 5,8
	n=3	n=3	n=3	n=3	n=4	n=4
96 h	99,4%± 1,04	95,3%± 4,11	98,4%± 2,77	93,1%± 1,86	96,8%± 2,42	89,67%± 3,37
	n=3	n=3	n=3	n=3	n=4	n=4 P=0,057

b

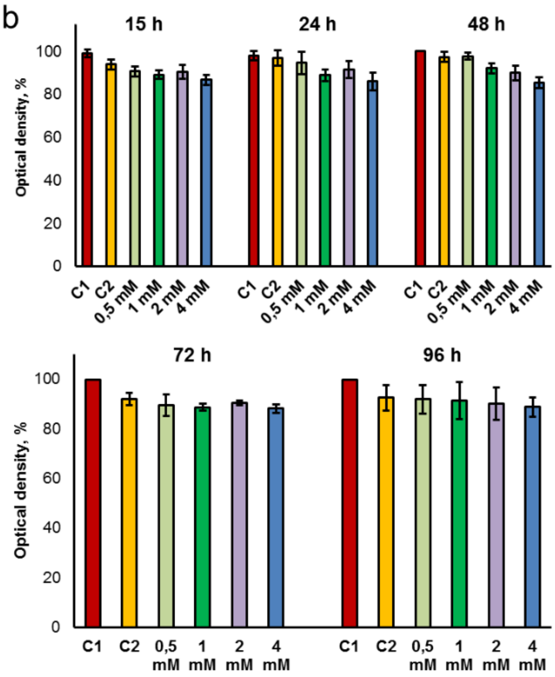

	C1	C2	0,5 mM	1 mM	2 mM	4 mM
15 h	99%± 1,73	93,87%± 2,27	90,57%± 2,23	89,03%± 1,94	90,4%± 3,07	86,77%± 2,32
	n=3	n=3	n=3	n=3 P=0,1	n=4 P=0,4	n=4 P=0,06
24 h	98%± 2,17	96,87%± 3,6	94,6%± 5,25	88,77%± 2,58	91,43%± 4,05	86%± 4,1
	n=3	n=3	n=3	n=3 P=0,1	n=4 P=0,23	n=4 P=0,057
48 h	99,97%± 0,06	97,33%± 2,39	97,63%± 1,63	92,03%± 2,4	89,93%± 3,47	85,37%± 2,36
	n=3	n=3	n=3	n=3 P=0,1	n=4 P=0,11	n=4 P=0,057
72 h	100%± 0	92,17%± 2,54	89,5%± 4,37	88,77%± 1,46	90,63%± 0,97	88,3%± 1,67
	n=3	n=3	n=3	n=3 P=0,1	n=4 P=0,57	n=4 P=0,2
96 h	100%± 0	92,63%± 5,2	92%± 5,71	91,5%± 7,37	90,2%± 6,55	88,83%± 4,01
	n=3	n=3	n=3	n=3	n=4	n=4

Figure S2. Evaluation of metabolic activity of human keratinocytes (**a**) and adenocarcinoma HeLa cells (**b**) with MTT assay**.** Both cell lines are treated with 0.5–4 mM of GA and incubated for 15 h and 24 h. C1 — cells growing in standard conditions (control); C2 — cells growing with ethanol as a solvent for GA (control for GA). The rate of metabolic activity after incubation with GA is compared with C2 samples. In each independent experiment (n), the maximal detected value of optical density is denoted as 100 %. The results on metabolic activity rate, standard deviation, number of independent experiments (n) and P-values are also presented in corresponding tables. Data are

shown as mean ± standard deviation (from 3–6 replicates of – 3-4 independent experiments (n)); * $p \leq 0.05$ according to the Mann — Whitney test.

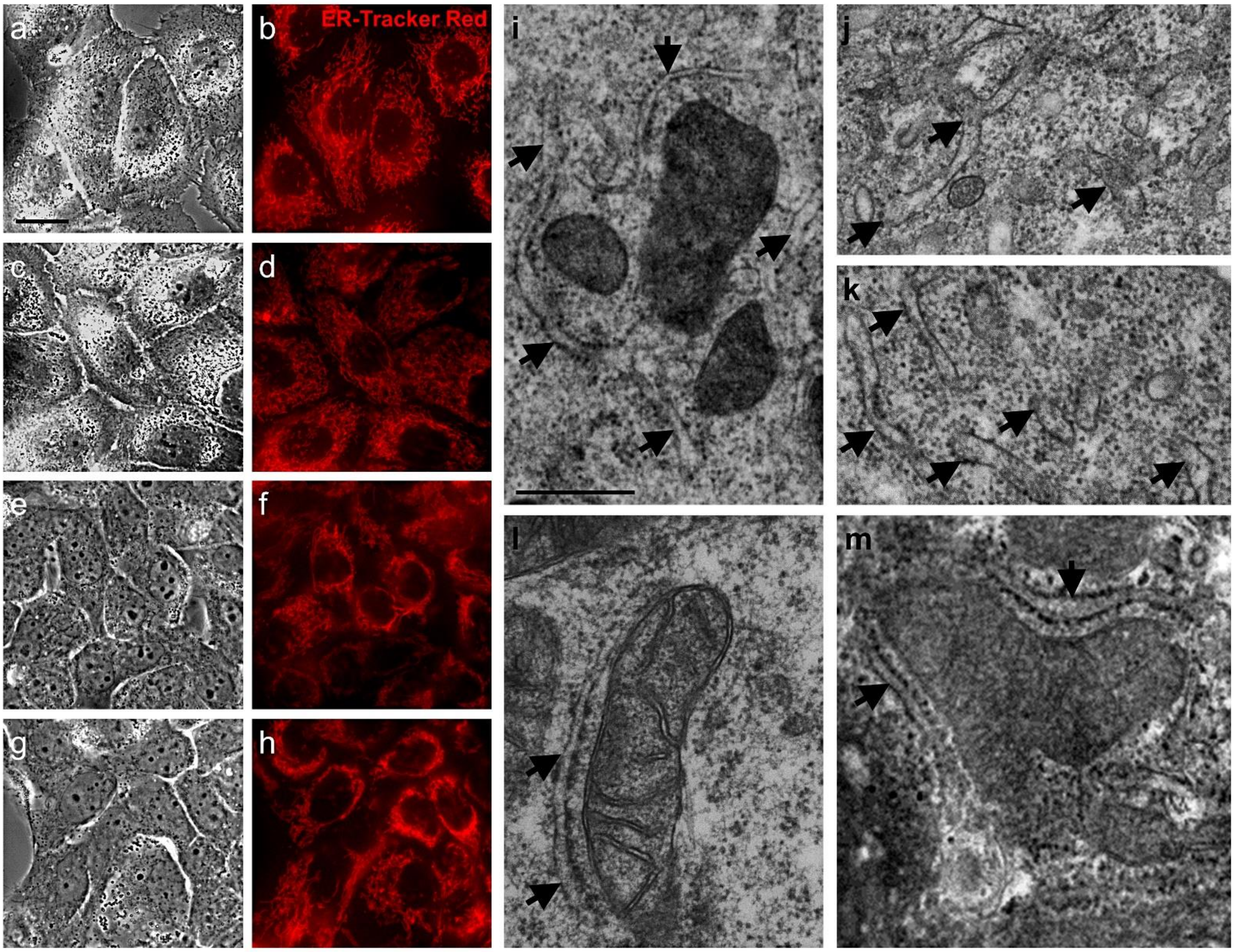

Figure S3. Visualization of localization and ER structure in keratinocytes HaCaT and carcinoma A431 cells using light and electron microscopy. (**a**, **b**) — HaCaT cells, control specimen; (**c**, **d**) — GA treated HaCaT cells; (**e**, **f**) — A431 cells, control specimen; (**g**, **h**) — GA treated A431 cells. Phase contrast images are shown on figures a, c, e, g; live staining with ER-Tracker Red is shown on figures b, d, f, h. The ultrastructural images of the ER cisternae in control specimen (**i**) and GA treated cells (**j**, **k**) HaCaT cells; the ultrastructural images of the ER cisternae in control specimen (**l**) and GA treated (**m**) A431 cells. Arrowheads indicate the ER cisternae covered with ribosomes. Scale bar for light microscopy images, 20 μm; for TEM images 0.5 μm.

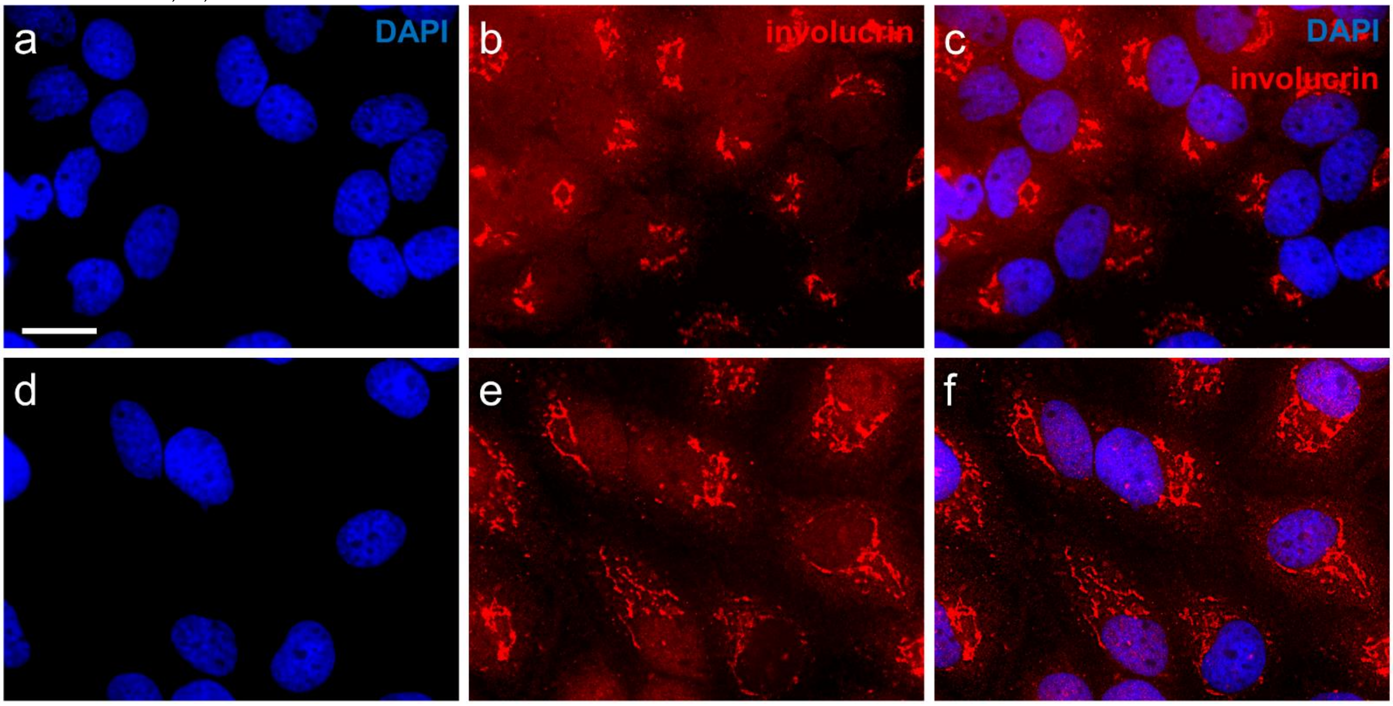

Figure S4. Immunocytochemical visualization of tubular/vesicular network with antibodies against involucrin in carcinoma A431 cells. (**a**–**c**) — control specimens, (**d**–**f**) — GA treatment. Nuclei are stained with DAPI (**a, d**), involucrin-positive staining is detected with anti-involucrin antibodies (**b**, **e**), merged images (**c, f**). Scale bar, 20 μm.

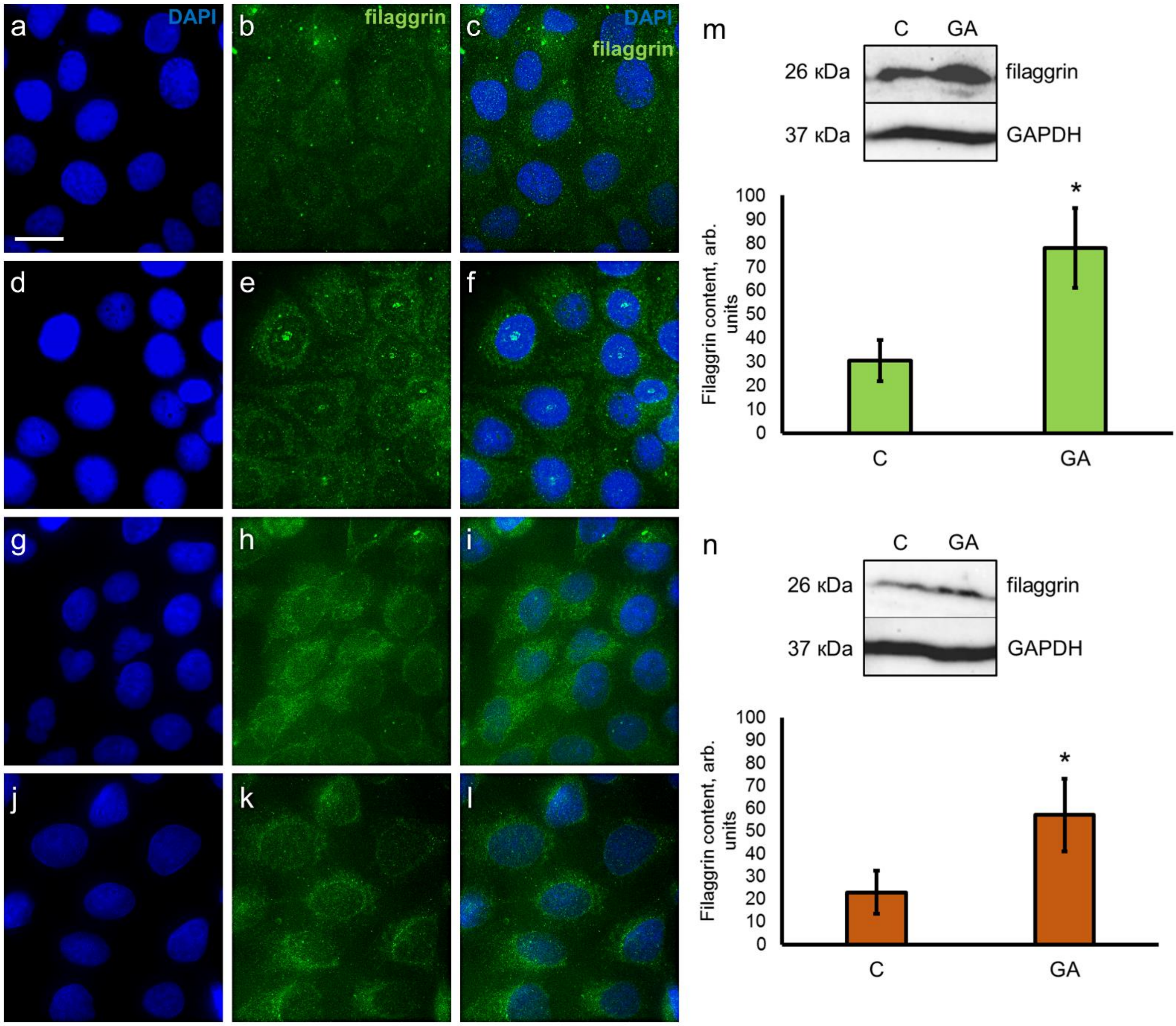

Figure S5. Immunocytochemical visualization and evaluation of filaggrin content in keratinocytes HaCaT and carcinoma A431 cells. (**a–f**) keratinocytes HaCaT in control specimens (**a–c**) and in the presence of GA (**d–f**); (**g–l**) carcinoma A431 cells in control specimens (**g–i**) and in the presence of GA (**j–l**). Nuclei are stained with DAPI (left column), filaggrin-positive staining is detected with anti-filaggrin antibodies (middle column), merged images (right column). Scale bar, 20 μm. Western blot analysis and evaluation of filaggrin content in HaCaT (**m**) and A431 cells (**n**). C — cells growing with ethanol as a solvent for GA (control for GA). Data are shown as mean ± standard deviation (n = 3); * $p \leq 0.05$ according to the Mann — Whitney test.
