# Supplementary material for: Gibberellic Acid Initiates ER Stress and Activation of Differentiation in Cultured Human Immortalized Keratinocytes HaCaT and Epidermoid Carcinoma Cells A431
Source: Pharmaceutics. 2021 Oct 30;13(11):1813. doi: 10.3390/pharmaceutics13111813 (PMC8622727; doi:10.3390/pharmaceutics13111813)
Supplement: Supplementary file 1 [file pharmaceutics-13-01813-s001.zip › Supplementary_Table S1_legend.pdf]

Table S1. The genetic information for HaCaT cell line. The data were provided by the Cell Culture Collection for Biotechnological and Biomedical Research, Koltsov Institute of Developmental Biology, Russian Academy of Sciences.

**Full genotype**		
AMEL	X	X
D3S1358	16	16
TH01	9,3	9,3
D12S391	18	23
D1S1656	11	12
D10S1248	14	15
D22S1045	15	16
D2S441	11	11
D7S820	9	11
D13S317	10	12
FGA	24	24
TPOX	11	12
D18S51	12	12
D16S539	9	12
D8S1179	14	14
CSF1PO	9	11
D5S818	12	12
vWA	16	17
D21S11	30,2	30,2
SE33	15	28,2

	**Genotype for search**	**Closets match**
**Cell Number**		**771**
**Cell name**		**HACAT**
**AMEL**	**X,X**	**X,X**
**D5S818**	**12,12**	**12,12**
**D13S317**	**10,12**	**10,12**
**D7S820**	**9,11**	**9,11**
**D16S539**	**9,12**	**9,12**
**vWA**	**16,17**	**16,17**
**TH01**	**9.3,9.3**	**9.3,9.3**
**TPOX**	**11,12**	**11,12**
**CSF1PO**	**9,11**	**9,11**
Comments	100 % match	
