# Supplementary figures and images for: Gibberellic Acid Initiates ER Stress and Activation of Differentiation in Cultured Human Immortalized Keratinocytes HaCaT and Epidermoid Carcinoma Cells A431
Source: Pharmaceutics. 2021 Oct 30;13(11):1813. doi: 10.3390/pharmaceutics13111813 (PMC8622727; doi:10.3390/pharmaceutics13111813)

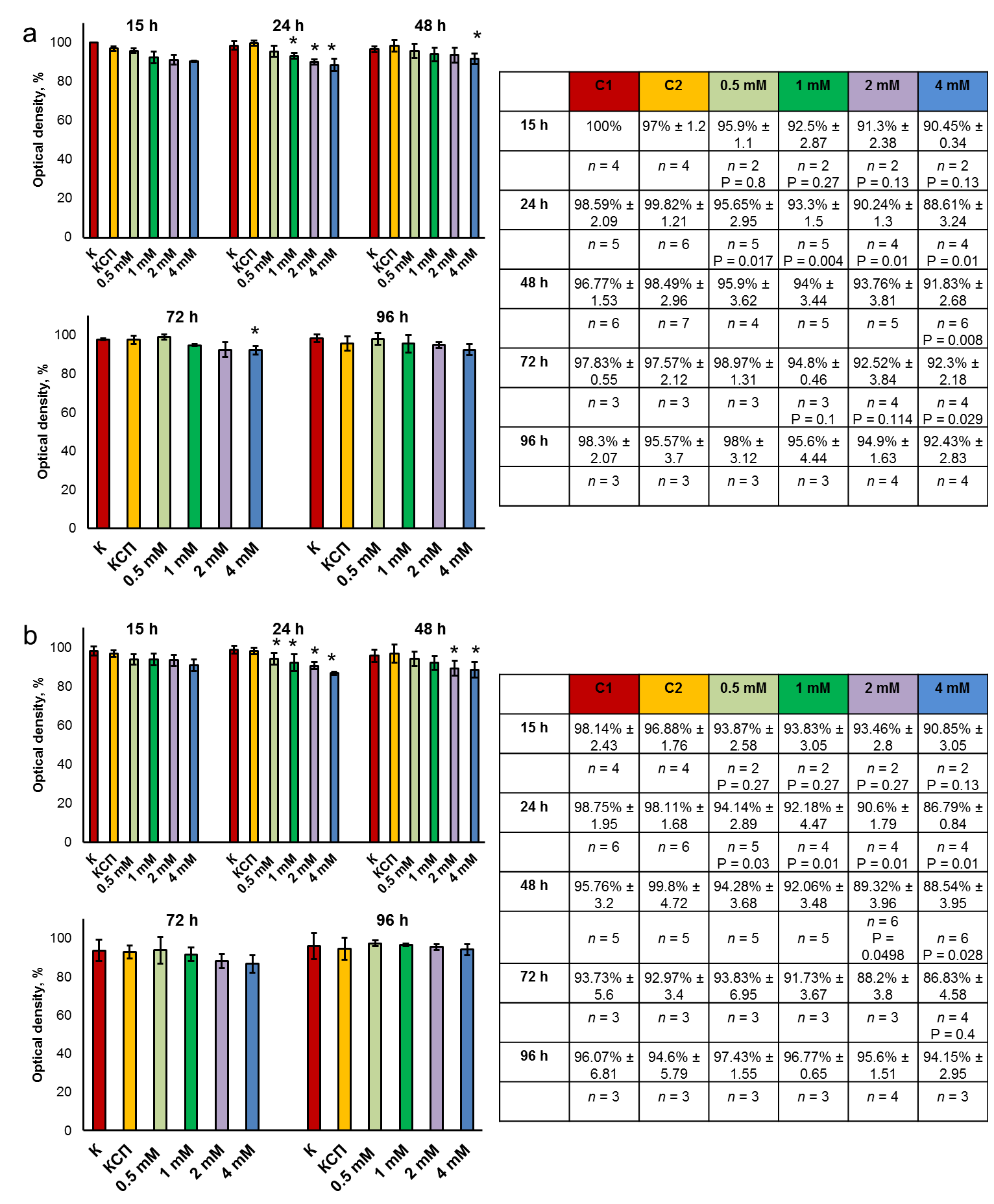

Supplement: Supplementary file 1 [file pharmaceutics-13-01813-s001.zip › Figure S1.tif]

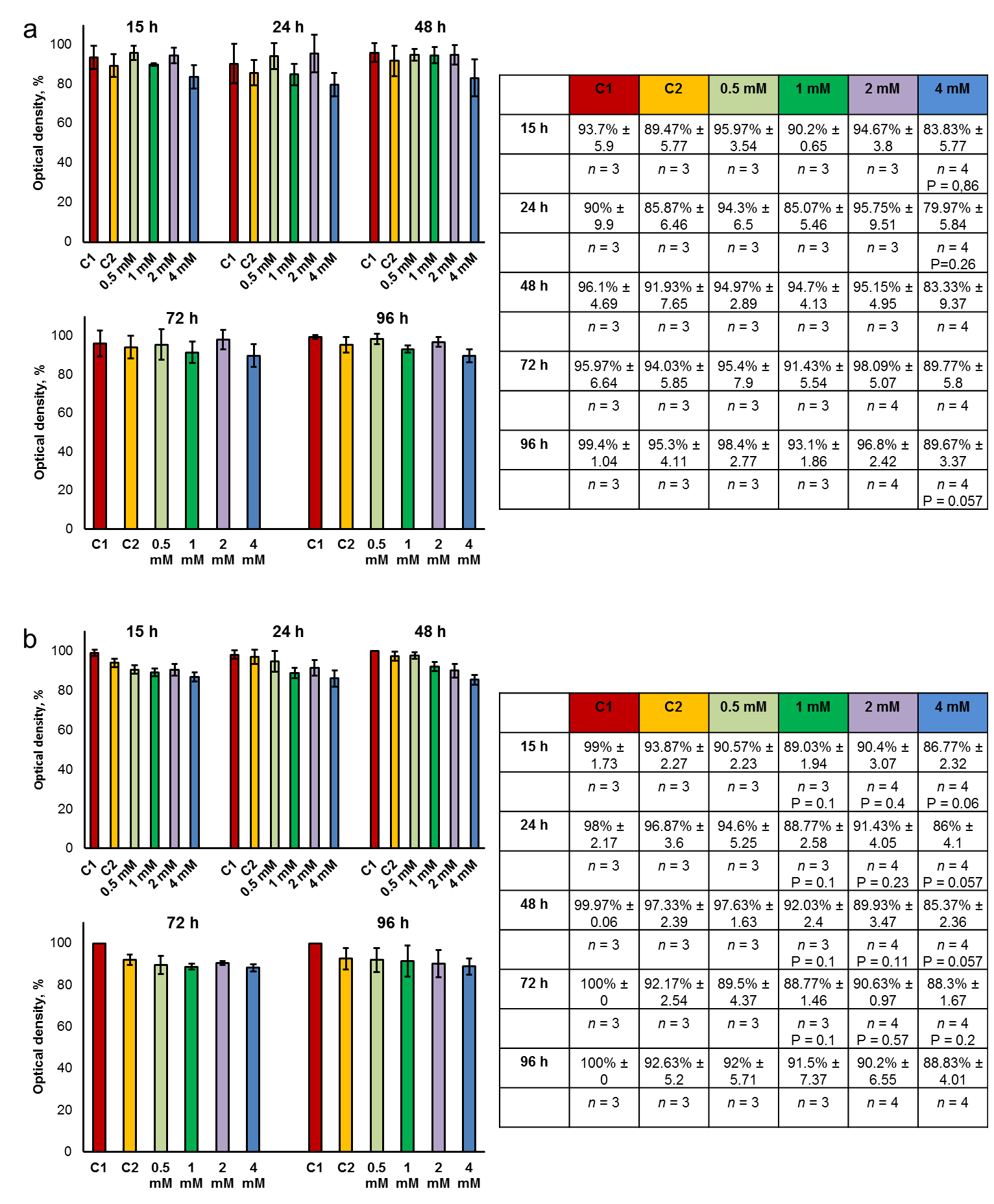

Supplement: Supplementary file 1 [file pharmaceutics-13-01813-s001.zip › Figure S2.tif]

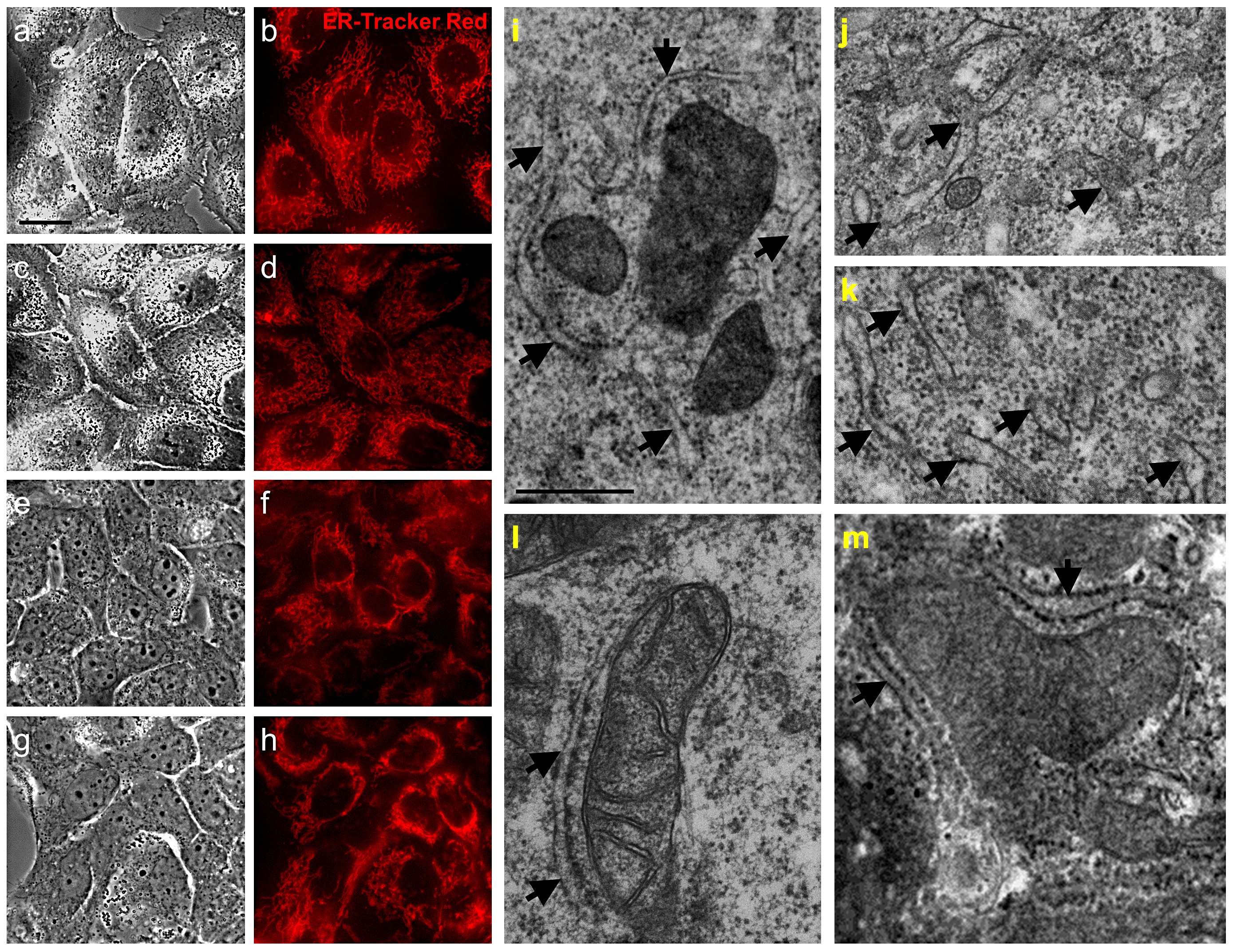

Supplement: Supplementary file 1 [file pharmaceutics-13-01813-s001.zip › Figure S3.tif]

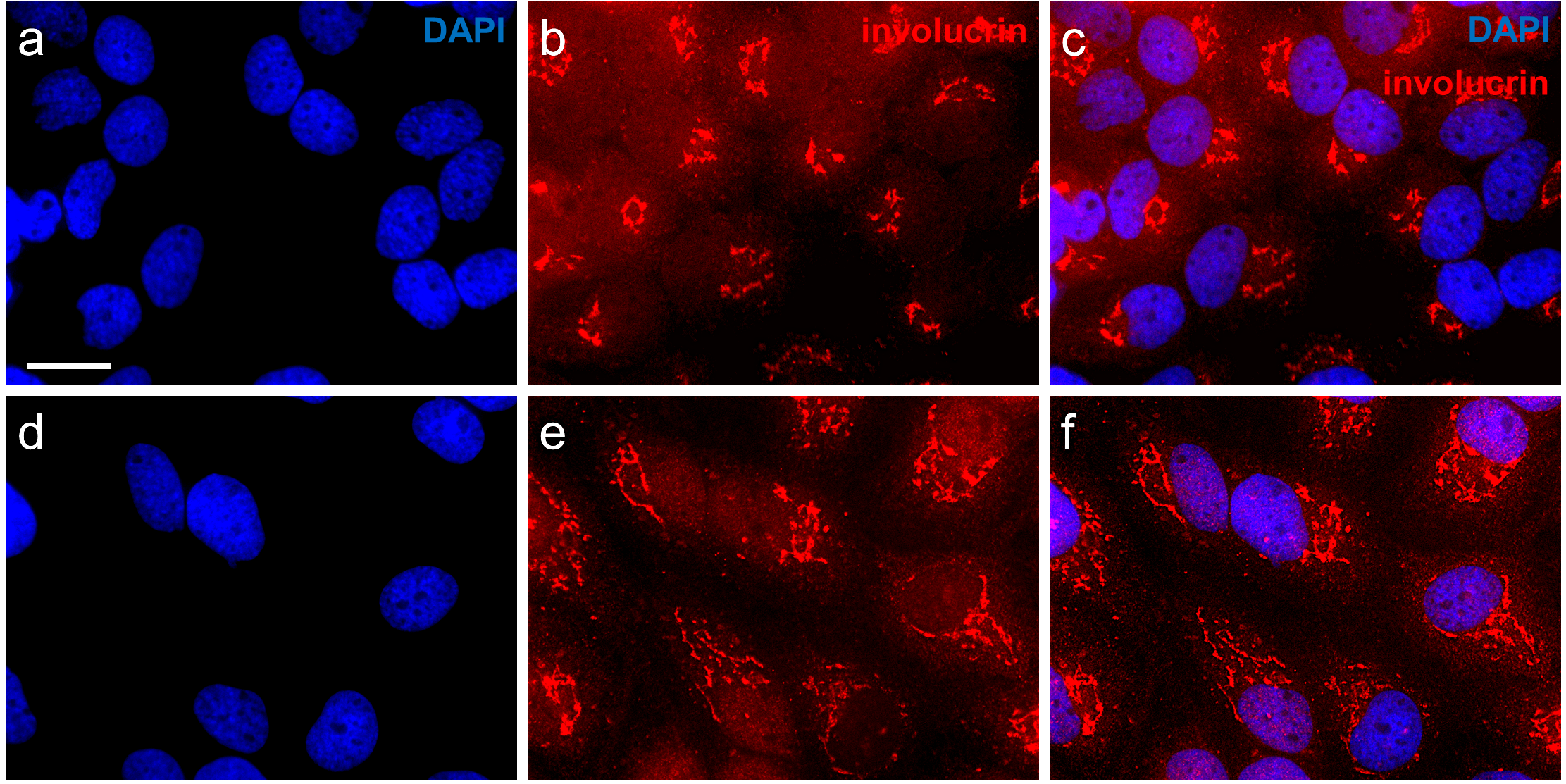

Supplement: Supplementary file 1 [file pharmaceutics-13-01813-s001.zip › Figure S4.tif]

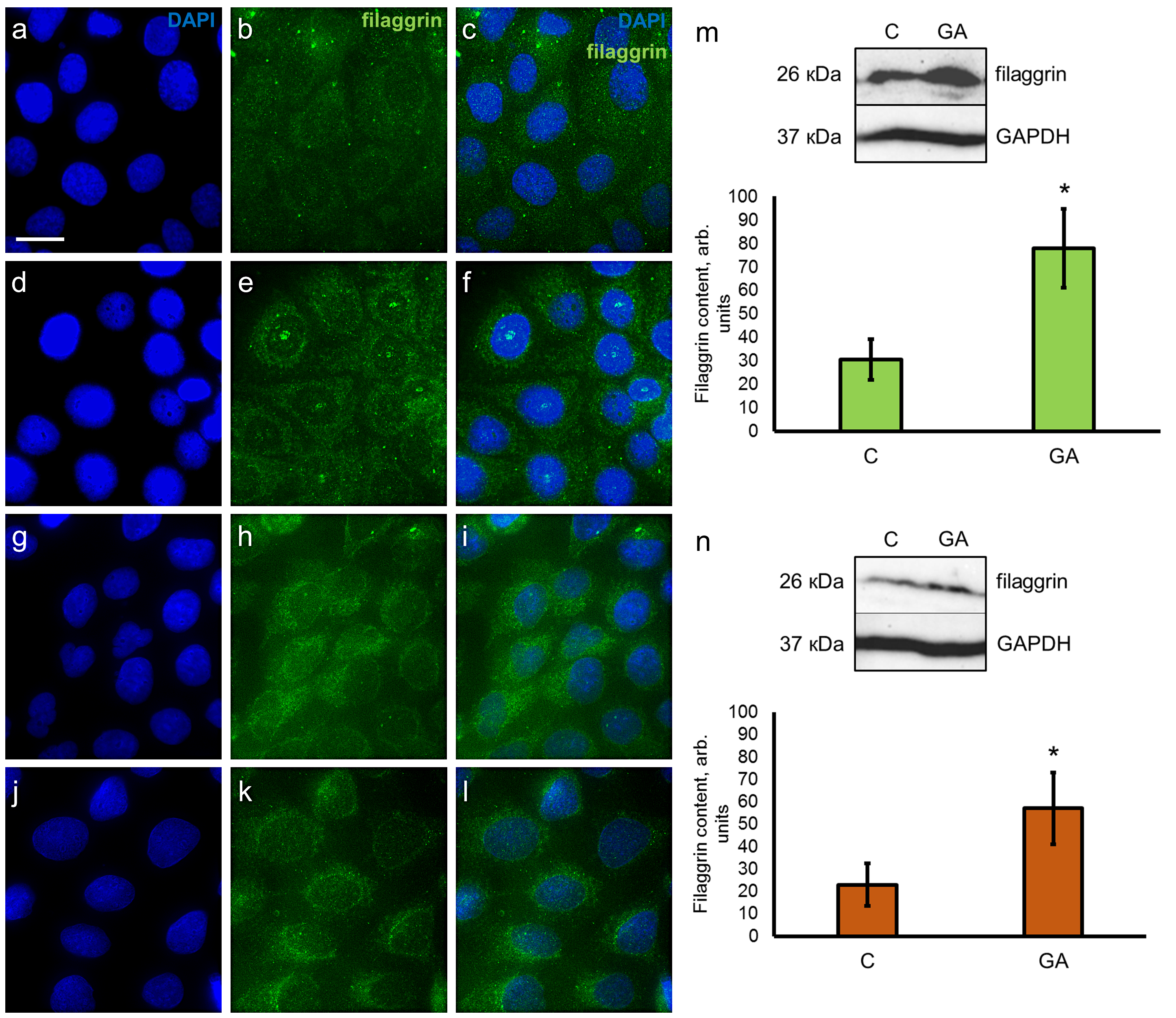

Supplement: Supplementary file 1 [file pharmaceutics-13-01813-s001.zip › Figure S5.tif]
